# Remote Consultations for People With Parkinson Disease and Cognitive Impairment: Interview Study With Patients, Caregivers, and Health Care Professionals

**DOI:** 10.2196/39974

**Published:** 2022-12-02

**Authors:** Jennifer Sarah Pigott, Megan Armstrong, Elizabeth Chesterman, Joy Read, Danielle Nimmons, Kate Walters, Nathan Davies, Anette Schrag

**Affiliations:** 1 Queen Square Institute of Neurology University College London London United Kingdom; 2 Centre for Ageing Population Studies Research Department of Primary Care and Population Health University College London London United Kingdom

**Keywords:** remote consultations, telehealth, telemedicine, Parkinson disease, cognitive impairment, Parkinson dementia, neurodegenerative condition, telephone appointments, video appointments, qualitative

## Abstract

**Background:**

The COVID-19 pandemic led to many consultations being conducted remotely. Cognitive impairment is recognized as a potential barrier to remote health care interactions and is common and heterogeneous in Parkinson disease. Studies have shown remote consultations in Parkinson disease to be feasible, but little is known about real-life experience, especially for those with cognitive impairment. We explored the experiences and perceptions of remote consultations for people with Parkinson disease and cognitive impairment.

**Objective:**

This study aimed to explore the experiences of remote consultations for people with Parkinson disease and cognitive impairment from the perspective of service users and professionals and investigate considerations for future service delivery.

**Methods:**

Semistructured interviews were conducted remotely with 11 people with Parkinson disease and cognitive impairment, 10 family caregivers, and 24 health care professionals (HCPs) between 2020 and 2021. Purposive sampling was used. Interviews were audio-recorded, transcribed, and analyzed using reflexive thematic analysis.

**Results:**

Overall, four themes were identified: “the nature of remote interactions,” “challenges exacerbated by being remote,” “expectation versus reality,” and “optimizing for the future.” Remote consultations were considered as “transactional” and less personal, with difficulties in building rapport, and considered to play a different role from that of in-person consultations. The loss of nonverbal communication and ability of HCPs to sense led to remote consultations being perceived as riskier by all groups. Issues arising from communication and cognitive impairment, balancing the voices of the person with Parkinson disease and the caregiver, and discussions of the future affect this population specifically. Remote consultations were reported to have been more successful than anticipated in all 3 groups. Obstacles were not always as expected; for example, age was less of a barrier than predicted. Video consultations were perceived as being preferable to telephone consultations by many participants, but not accessible to all people with Parkinson disease. With widespread expectation of ongoing remote consultations, potential improvements for these 3 groups and health care services were identified, including practice, preparation, increased awareness of issues, expectation management by HCPs, and more time and flexibility for consultations.

**Conclusions:**

Advantages and challenges of remote consultations for this population are identified. Consultations could be improved with increased support, practice, preparation, awareness of issues, and more time and flexibility within services.

## Introduction

### Background

On declaring COVID-19 as a pandemic in March 2020, the World Health Organization advocated strict social distancing and quarantine measures to avoid virus spread [1]. Health services rapidly used telemedicine to deliver care for many conditions, including Parkinson disease [2-6]. Telemedicine is the delivery of health care services, where distance is a critical factor, using information and communication technologies [7].

Parkinson disease is a heterogeneous neurodegenerative condition, affecting >6.1 million people globally, with rates rising [8]. People with Parkinson disease frequently experience cognitive symptoms [9], with impairment increasing with age and duration of disease: 80% of people with Parkinson disease have dementia by 20 years of disease duration [10].

Remote consultations (telephone or video call) are not completely new. Studies have shown feasibility of specialist consultations and delivery of therapy for people with Parkinson disease [11-15], and high rates of interest in telemedicine among people with Parkinson disease have been reported [16,17]. Patient perspectives have tended to be explored within research contexts, a qualitative survey within a US-based trial of Parkinson disease specialist internet-based visits collated feedback from specialist and patient participants [18]. They identified positive and negative perceptions driven by three themes: personal benefits (eg, lack of travel and frustration), perceived quality of care (eg, more frequent visits and technical troubles), and quality of interpersonal engagement (eg, liked the physician and difficult communication). Studies of remote consultations in Parkinson disease have predominantly been undertaken with educated, digitally literate samples, with digital resources provided and technical support available; therefore, little is known about real-life use. A recent study of neurologists’ experiences of remote consultations (not Parkinson disease–specific) found perceived improved access and efficiency and an expectation that telemedicine will continue to be used beyond the pandemic. However, in-person consultations were not felt to be fully replaceable owing to great uncertainty when working remotely, technical and administrative problems, and “difficult consultations.” Consultations reported as “difficult” included those with new patients and those with cognitive impairment and consultations requiring difficult decisions or significant discussions (eg, breaking bad news) [4]. A recent qualitative study exploring the impact of the COVID-19 pandemic on Parkinson disease management, from the perspective of people with Parkinson disease and health care professionals (HCPs), reported mixed reactions to remote consultations [19]. Most study participants were able to use internet-based technologies, which the authors acknowledged may be unrepresentative of the wider older population living with Parkinson disease, and cognitive impairment was not explored.

A qualitative study exploring the experiences of remote consultations for people living with non-Parkinson dementia and their carers identified various difficulties: the lack of prompts to remember problems, dealing with new emerging difficulties, rescheduling or missed calls, and inclusion of the voice of the person with dementia [20]. However, to the best of our knowledge, no studies have investigated remote consultations for people with Parkinson disease who have cognitive impairment. The combination of physical and cognitive impairments and the pattern of cognitive deficits in Parkinson disease differs from other types of dementia [21-24], which may convey different experiences and needs.

### Objective

This study aimed to explore the experiences of remote consultations for people with Parkinson disease and cognitive impairment and investigate considerations for future service delivery.

## Methods

### Design

An exploratory qualitative design using semistructured interviews, analyzed using thematic analysis, with reporting guided by the Standards for Reporting Qualitative Research framework [25].

### Ethics Approval

This study was approved by the London Queen Square Research Ethics Committee (18/LO/1470).

### Sample and Recruitment

Overall, three groups of participants were recruited between October 2020 and July 2021: people with Parkinson disease and cognitive impairment, family caregivers, and HCPs working with this group. People with Parkinson disease and caregiver participants were purposively sampled to ensure representation of different clinical and social backgrounds in terms of age, ethnicity, education, living arrangements, duration of disease, and severity of impairments (functional and cognitive) managed through different health care services. Potential participants were identified through clinicians in primary and secondary care, or participants self-presented to the research team following charity sector advertisement. Additional recruitment sites were approached in more ethnically diverse areas to try to improve recruitment from ethnic minorities.

For HCPs, a range of different professional backgrounds was sought, working within different geographical areas and services, with a variety of experience of remote consultations. HCP participants were also identified through snowballing, using professional networks. HCP participants needed adequate experience of working with people with Parkinson disease to recall clinical encounters for discussion, but a range of expertise was sought. To represent the broad range of disciplines involved in the care of people with Parkinson disease [26], greater numbers of HCPs compared with people with Parkinson disease and caregivers were required. All potential participants were screened for eligibility using inclusion and exclusion criteria presented in Textbox 1 and sent detailed information via post or email. All participants provided formal consent, in the form of written, digital, or audio-recorded verbal consent.

**Inclusion and exclusion criteria.**

**Inclusion criteria for people with Parkinson disease and cognitive impairment**

- Diagnosis of idiopathic Parkinson disease made by a clinical specialist
- Cognitive symptoms, based on subjective report (participants reporting subjective cognitive symptoms, identified by a clinician as having cognitive impairment, were included even in absence of formal diagnosis because cognitive symptoms are common, but often missed in clinical practice [27]. Participants were not included if they denied cognitive symptoms despite a clinician identifying them, because it would not be appropriate to attempt detailed interview discussion of these symptoms with them)—described in lay terms as “changes in memory, thinking, concentration”

**Exclusion criteria for people with Parkinson disease and cognitive impairment**

- Care home residents
- Individuals with atypical Parkinsonian disorders
- Individuals anticipated to be approaching end of life

**Inclusion criteria for caregivers**

- A person who closely supported the person with Parkinson disease (person being supported must meet inclusion criteria mentioned previously)

**Inclusion criteria for health care professionals**

- A person working within or in collaboration with health care, who encounters people with Parkinson disease and cognitive impairment in a professional capacity

### Data Collection

Topic guides were designed following a review of the literature and refined with multidisciplinary and patient and public involvement (PPI) input, to explore experiences and perceptions of remote interactions for health and social support. Interviews were conducted by JP (a geriatrician trained in qualitative research methods), via either telephone or video call. Interviews were audio-recorded and transcribed. Data collection continued until the team was confident that the breadth of representation and the depth of information obtained was sufficient to address the study’s aim.

### Analysis

Interviews were transcribed verbatim and analyzed using reflexive thematic analysis within a predominantly experiential qualitative framework [28,29]. The coding framework was generated following discussions among the team members, who had read a sample of transcripts; revised iteratively as it was applied to remaining transcripts (JP and EC); and presented for wide team review. Line-by-line coding was conducted using NVivo (version 12; QSR International) [30]. All extracts assigned to each code were reviewed and grouped to organize themes and further refined through discussion and interpretation with the research team and PPI. The multidisciplinary team included those with background in geriatric medicine (JP), nursing (EC and JR), psychology (MA, ND, and JR), neurology (AS) and general practice (DN and KW).

## Results

### Overview

In total, 11 people with Parkinson disease, 10 caregivers, and 24 HCPs were interviewed. Overall, 5 interviews were conducted as people with Parkinson disease–caregiver dyad interviews, with 10% (1/10) of the caregivers subsequently also interviewed alone. In total, 5 individual caregiver interviews were conducted where the people with Parkinson disease felt unable to participate. Overall, 47% (8/17) of interviews with people with Parkinson disease and caregiver were conducted via video call and 53% (9/17) via telephone call, with duration ranging from 41 to 121 minutes. Of the 24 HCP interviews, 21 (88%) were conducted via video call, 2 (8%) were via telephone call, and 1 (4%) was in person, with duration ranging from 41 to 98 minutes. All people with Parkinson disease (11/11, 100%) and caregiver participants (10/10, 100%) were from the Southeast or East of England; HCPs were from the Southeast of England, the Midlands, and Scotland. Demographic details about the participants are presented in Tables 1 and 2.

**Table 1:** Demographic details for people with Parkinson disease and caregivers.

Group and characteristics				Participants
**People with Parkinson disease represented by interviews with people with Parkinson disease and caregivers (n=15)**				
	Age (years), mean (SD)			75.7 (8.2)
	**Sex, n (%)**			
		Male	9 (60)	
		Female	6 (40)	
	**Ethnicity, n (%)**			
		White—British	12 (80)	
		White—other	1 (7)	
		Asian—Indian	1 (7)	
		Black—other	1 (7)	
	Duration of Parkinson disease (years), mean (SD); range			13.6 (6.7); 2-25
	**Cognitive impairment, n (%)**			
		Subjective symptoms, without formal diagnosis (varying severity)	8 (53)	
		Existing diagnosis of mild cognitive impairment	1 (7)	
		Existing diagnosis of dementia^a^	6 (40)	
	**Educational background^b^**			
		Age at leaving full-time education (years), range	14-25	
	Schwab and England scale [31]^c^ (%), mean (SD); range			47.5 (30); 10-100
	**Living arrangements, n (%)**			
		Live with spouse or partner	6 (40)	
		Live with family	4 (27)	
		Live alone	5 (33)	
	**Location, n (%)**			
		Urban or suburban	13 (87)	
		Semirural	1 (7)	
		Rural	1 (7)	
**Caregivers (n=10)**				
	**Relationship, n (%)**			
		Spouse	5 (50)	
		Daughter	5 (50)	
	Age (years), mean (SD); range			62.8 (11.1); 46-78
	**Sex, n (%)**			
		Male	3 (30)	
		Female	7 (70)	
	**Ethnicity, n (%)**			
		White—British	8 (80)	
		Asian—Indian	1 (10)	
		Black—Caribbean	1 (10)	

^a^Of the 6 participants, 2 (33%) were interviewed directly, and for the remaining 4 (67%), interviews were conducted with caregiver only.

^b^Qualifications range from none through to degrees.

^c^Indicates degree of impairment, with 100% being independent and 0% being fully dependent.

**Table 2:** Roles of health care professional participants (n=24).

Professional role	Participants, n (%)
Parkinson disease nurse specialist	4 (17)
Neurologist	3 (13)
Geriatrician	3 (13)
General practitioner	3 (13)
Clinical neuropsychologist	2 (8)
Speech and language therapist—neurology services	2 (8)
Occupational therapist—memory service	1 (4)
Occupational therapist—movement disorders service	1 (4)
Physiotherapist—movement disorders service	1 (4)
Older adult psychiatrist	1 (4)
Mental health nurse—memory service	1 (4)
Palliative care physician	1 (4)
Charity sector—Parkinson’s UK local advisor^a^	1 (4)

^a^Charity sector role to help people with Parkinson disease, including providing advice and information and supporting access to services.

Participants described the uses of remote communication technology in different aspects of their lives. All people with Parkinson disease and caregiver participants used telephones for personal communications; several of them had used video calls socially in the past, and all of them had used it during the pandemic. All people with Parkinson disease and caregivers had experienced telephone consultations, but only few of them had experienced video consultations; thus, they spoke about their experience of video technology in general. HCPs’ experience of video consultations was varied, with most consultations conducted via telephone (experienced by all; 24/24, 100%). Although not the focus of discussion, some participants described the use of asynchronous email or SMS text message communication. All remote consultations had been a result of the pandemic, with a few now expressing it as a preference. Several caregivers for people with Parkinson disease with severe impairments explained that the people with Parkinson disease could not use the telephone or video themselves. All people with Parkinson disease and caregiver participants had established Parkinson disease; HCPs recalled experience of both new and established patient encounters. The interview discussions led to four themes: “the nature of remote interactions,” encompassing subthemes “a transactional exchange,” “is it real?” and “a risky process”; “challenges exacerbated by being remote,” encompassing subthemes “communication and understanding,” “interpersonal dynamics,” and “significant discussions”; “expectation versus reality,” encompassing subthemes “anticipated barriers” and “expected advantages”; and “optimizing for the future,” encompassing subthemes “support for people with Parkinson disease and cognitive impairment and caregivers,” “professional development,” and “service improvement” (Textbox 2). Additional quotes from participants are provided in Multimedia Appendix 1.

**Themes and subthemes.**

**Theme 1**

- The nature of remote interactions
- Subthemes
  - A transactional exchange
  - Is it *real*?
  - A risky process

**Theme 2**

- Challenges exacerbated by being remote
- Subthemes
  - Communication and understanding
  - Interpersonal dynamics
  - Significant discussions

**Theme 3**

- Expectation versus reality
- Subthemes
  - Anticipated barriers
  - Expected advantages

**Theme 4**

- Optimizing for the future
- Subthemes
  - Support for people with Parkinson disease and cognitive impairment and caregivers
  - Professional development
  - Service improvement

### The Nature of Remote Interactions

Differences in the interaction via remote technologies were described, typically regarding the lack of physical contact (including examinations) and visual information and cues. The consequences are encompassed by three subthemes: “a transactional exchange,” “is it real?” and “a risky process.”

#### A Transactional Exchange

Participants described the “transactional” nature of remote consultations. Most participants, across the groups, felt that building rapport was more difficult remotely, which is exacerbated by technical issues. Some felt this improved over time with multiple consultations and with video over telephone. Many HCPs found it more difficult to manage people with Parkinson disease and caregiver emotions and offer reassurance remotely, for both video and telephone consultations. Many participants, particularly people with Parkinson disease, perceived the consultation as more automatic and functional, with less personalization:

I don’t always feel that there is a proper dialogue. It’s a question-and-answer sort of thing that goes on. But it sounds a bit mechanical. Sort a list of things to tick off.

Person with Parkinson disease 1

HCPs often attributed the dynamic to the lack of physical contact or visual interaction or environmental factors, affecting both telephone and video consultations, but more so with telephone consultation:

I think when they’re with you in a room and they feel safe in that environment then they will talk to you more.

HCP 25; occupational therapist; OT

In contrast, people with Parkinson disease and caregivers were more likely to attribute this to the clinician’s approach and style of questioning (such as checklists). They felt more rushed, thus sensing they were a burden:

I think it removes some of the pastoral nature of the role, because it feels like you’re just taking up their time.

Caregiver 2

HCPs reported using techniques such as checklists and closed questioning, trying to overcome the difficulties of assessing remotely:

I have found a checklist works really well, because when you’re trying to juggle the phone and the video or whatever, knowing I’m going through a checklist and I know where I am.

HCP 19; neurologist

Several participants reflected on a nebulous concept of human interaction, “hard to put into words*”* that is lost remotely, whether telephone or video consultation, leading to less “enjoyable” or “fulfilling” interactions. It is something more than just visual, related to “more dimensions of engagement” (HCP 24; palliative care physician) with physical presence. This affected satisfaction across participant groups.

#### Is It Real?

Some participants perceived remote consultation to take a different role than in-person consultation, with some HCPs observing that people with Parkinson disease did not “count” remote consultations, *“*they don’t see it as a consultation*”* (HCP 21; Parkinson disease nurse specialist), but rather perceived them as an informal “chat” or “check-in,” in some cases, as a “stepping-stone” to in-person consultation. This impression was substantiated across all groups by participants’ language, contrasting remote consultations to “real life” (HCP 10; geriatrician) or referring to in-person consultations as being “properly seen” (caregiver 14). This was reported for both telephone and video consultations, but more emphasized for telephone consultations. Consequences of this were the impact on the professional-patient relationship. HCPs implied that they detected less respect for remote consultations:

But patients will say, “Yes, yes, that’s fine. I can do that,” and then they don’t turn up [for the video call]. And I find they haven’t even bothered to try. They’ve gone in the garden because, actually, it just seemed like too much bother.

HCP 27; physiotherapist

In contrast, a sense of distrust emerged from some people with Parkinson disease and caregivers:

They didn’t say they got it [prescription] wrong. But I still don’t know whether they, they had got it wrong. So there’s that element in view of the virus, doing it all from arm’s length...if I’m being honest, I wasn’t totally sure that they were being that straight with me.

Person with Parkinson disease 1

#### A Risky Process

Participants from all 3 groups spoke of deficiencies in remote consultations, both telephone and video consultations, that generated anxiety. Several HCPs were concerned about the medicolegal standing and “unintentionally being negligent*”* owing to lack of “standardized procedure” (HCP 24; palliative care physician). Increased risk was described in relation to perceiving a high chance of error. HCPs universally reported difficulty in making assessments without the usual information, frequently citing the importance of physical examination or observing task performance for Parkinson disease and cognitive assessments, particularly in diagnostic contexts. Several participants were concerned about not getting the “full picture” remotely, where during in-person consultations, they would rely on different information streams (eg, verbal and nonverbal cues, observation, and examination) especially for complex cases. This could be moderately alleviated by good quality video consultations, but observation via video was frequently inadequate, and it still lacked hands-on examination. Some participants elaborated further, describing reliance on a “sense” for clinical judgments when in person:

As psychologists there is a lot of, you know, you can feel from people, you know, there is, kind of, actually, “I feel that you seemed quite upset when I said that,” and that’s sometimes difficult to do over Near Me [video conferencing] apparatus, as well. So, it’s the kind of, non-spoken subtleties I think that you miss sometimes over the technology.

HCP 18; neuropsychologist

All participant groups were concerned that impairments could be concealed in remote consultations, which may have been identified in person. From the people with Parkinson disease and caregiver perspective, there was a sense of unease about HCP judgments relying on their symptom descriptions during telephone calls:

...Sometimes you get a doctor who I’ve never met, and you’re talking to you over the phone. They’ve never met my father, and it’s, it just feels a bit tenuous. Can you – can you really? It feels, it’s too much responsibility to me. Have I described everything?

Caregiver 2

Further risk related to who is present for remote consultations: both expressing concern if consultations were unsupervised and the presence of unknown others (not visible during telephone consultation and out of view during video consultation). HCPs reported that people with Parkinson disease were potentially exposed to physical risk while performing assessment tasks or emotional vulnerability when discussing sensitive topics, if they are alone:

There have been occasions where patients with low mood do, kind of, talk about suicidal thoughts and things like that, in the hospital environment it feels safe enough to discuss those sorts of things, whereas, when you’re not with the patient I wouldn’t feel comfortable about those kinds of things with them.

HCP 25; OT

Some participants from each group questioned digital security, nonprivate health care work environments, and confidentiality with others on the call:

I just think that everyone seems to be talking at once at all times and you don’t know who you’re talking to as a GP, and it makes me feel a bit uncomfortable like who actually is in the room.

HCP 13; general practitioner; GP

### Challenges Exacerbated by Being Remote

Participants described challenges in health care interactions driven by the condition, many of which were exacerbated by being remote. They were grouped into three subthemes: “communication and understanding,” “interpersonal dynamics,” and “significant discussions.”

#### Communication and Understanding

The dual impact of physical (eg, quiet speech) and cognitive (eg, difficulty in multitasking and memory problems) symptoms of Parkinson disease impeded communication, sometimes compounded by, for example, hearing impairment. They led to problems for people with Parkinson disease in understanding and retaining information or instructions. HCPs described frustration at not being able to physically *show* people with Parkinson disease what to do or give hard copies of information leaflets as they would in person. These communication difficulties were felt to be even more challenging remotely, owing to unfamiliarity with technology for video and reliance on verbal communication for telephone:

I hate using the phone. I get on the phone and then I don’t understand people.

Person with Parkinson disease 6

Some participants from each group described people with Parkinson disease finding it more difficult to keep up with conversation over remote communication methods (both video and telephone) owing to slowed speech, slowed processing, and forgetting:

He can’t really remember what’s been said, so he finds it difficult to process the information. So, having a telephone conversation with him is even more difficult than a face-to-face conversation.

Caregiver 12

Difficulties in sustaining engagement, perhaps related to concentration or apathy, were worse remotely owing to additional distractions and lack of eye contact. The pace of conversation needed to be slower. Breakdown of video feeds owing to unstable connections could interfere with communication and telephone pauses could be misinterpreted owing to lack of visual cues:

On the phone the other day there were these silences and I was thinking, have they not heard, are they shaking their heads or are they tutting, what’s going on at the other end, you know, I had no idea, it was a bit unsettling.

HCP 17; geriatrician

For all types of remote consultation, the lack of usual contextual cues could lead to increased disorientation for the people with Parkinson disease—several HCPs described people with Parkinson disease forgetting the purpose of a call or who they were. The cognitive burden, and in some cases, associated anxiety, of remote consultations, particularly video consultations, was typically perceived as greater:

If there is cognitive impairment that’s massive, actually, yes, that’s quite a big deal because, again, the multiple stimuli that you have can confuse the conversation.

HCP 24; palliative care physician

However, this was not universal—a few participants described finding the familiarity of their own environment more relaxed and conducive to remembering and understanding:

You’re in your own comfort zone and you absorb it better than what you do when you have to travel.

Caregiver 3

#### Interpersonal Dynamics

Although similar to in-person appointments, the additional communication and technical challenges of remote consultations led to increased need for people with Parkinson disease who had cognitive impairment to have caregiver support. In many cases, there was increased reliance on family or friends beyond a spousal care partnership to use remote technologies because caregivers also had difficulties. Many participants found that these increased support needs led to great tendency to exclude the person with Parkinson disease, either through the consultation being solely with the caregiver or the person with Parkinson disease being *spoken for* within a joint consultation:

I think the patient is a bit more cut out, and I’m aware of that, that when they’re in the clinic and I talk to both, it’s a bit more the carer but the patient is still there.

HCP 19; neurologist

Many participants appeared dissatisfied with this shift in dynamic. At times, it was implied or requested by the people with Parkinson disease, but by and large, it appeared to be automatic, that is, from perceived necessity:

...It’s quite hard, because sometimes I feel like I could take over from it. I try not to; I try to get her to explain herself, but she does – I feel like she’s not explaining herself properly. So I end up, OK, then I’ll explain what I’ve seen to the doctor.

Caregiver 15

#### Significant Discussions

Diagnoses and prognoses were considered as potentially difficult conversations for HCPs delivering them and for people with Parkinson disease and caregivers receiving them. There was universal agreement that these should be conducted in person rather than remotely. Discussions about progression, the future, and advanced care planning were perceived by HCPs as difficult but important topics, particularly in this population. Most HCPs found them to be even more challenging through remote interactions:

It [talking about the future remotely] probably takes longer, in that people- it’s probably slightly more intense, you can’t soften it as much. Being in person you can probably soften those conversations a bit more and make them slightly less stark.

HCP 8; GP

The difficulties may even prevent them from being held:

I’ve been terrible at doing it [advanced care planning].

HCP 14; neurologist

Participants from all groups indicated that people with Parkinson disease and caregivers may feel less confident or secure to ask about the future in a remote consultation, with a few participants feeling that video consultation was marginally less of a barrier than telephone consultation:

Yeah...not on the phone I think...I think it’s having the confidence to speak to them and if I’ve got any questions and the thought of really having something bad going on in your head, that’s, that’s the frightening bit.

Person with Parkinson disease 3

### Expectation Versus Reality

With the rapid implementation of remote consultations owing to the COVID-19 pandemic, many participants reflected on what they had expected the experience to be compared with the reality. This is encompassed by two subthemes: “anticipated barriers” and “expected advantages.”

#### Anticipated Barriers

Although participants reported their experiences critically by reporting challenges, most participants actually indicated being “surprised” at how well remote consultations had been experienced. They reported it being easier and more similar to in-person consultation than expected, for video consultation and even telephone consultation:

In some respects that’s exactly what we would be doing when we saw them face-to-face.

HCP 5; OT

Across all groups, many participants anticipated older age to be a barrier to video consultations, but this was often not the case. Some HCPs indicated that older people with Parkinson disease had more reservations or difficulties with the technology, but most of them thought that the barrier was lack of experience or personality rather than age. The reported use of technology by the people with Parkinson disease and caregiver participants also suggested that familiarity was more relevant than age:

If it’s not someone who’s familiar with a computer, an iPad, for example, then it’s all new learning and it’s quite a lot of ask. But, if somebody is familiar with it and has been using it during their life, which lots of people have and do, irrelevant of age, actually, then there’s a bit of that information already there.

HCP 9; neuropsychologist

However, there were some descriptions of remote technology being embraced more by young generations, owing to convenience:

It suits working people that they can just duck out, make a phone call and then they can go back to work.

HCP 15; GP

In addition, even if people with Parkinson disease owned and were familiar with digital devices, they may be anxious:

The fact is that they haven’t got the confidence to press that button.

HCP 12; Parkinson’s UK advisor

Cognitive impairment was not a universal barrier to using remote technology, but use rather depended on the degree of impairment and support provided. Difficulties with technology were reported across the participant groups, likely related to cognition. All except the people with Parkinson disease with severe impairment appeared to be able to undertake telephone consultations (some requiring support), but HCP participants had found that video consultations were less accessible for this population, and people with Parkinson disease and caregiver participants reported barriers to use of video calls in their personal lives. For some people with Parkinson disease, cognitive impairment prevented new learning, and even some individuals with past experience had lost their technological capability:

I just find anything I do, on a laptop or a computer, never seems to work out the way it’s supposed to.

Person with Parkinson disease 4

Several issues that were described, such as lack of visual and touch information, although perceived as challenging, were not *as* restrictive as had been anticipated; more could be achieved remotely than expected. Universally, discussing potentially sensitive symptoms (eg, bowels or sexual function) remotely was not considered problematic:

I mean sensitive is sensitive.

Person with Parkinson disease 1

Ease of discussion was more dependent on the individuals involved, their relationship, and manner in which it was approached, rather than method of consultation; however, some topics, particularly mental health, were more difficult, typically owing to lack of rapport. However, across the groups, a few participants expressed opposing views, finding the remoteness helpful for sensitive topics:

I feel I can have quite probing conversations and not feel awkward. So maybe for me that layer of the subconscious awkwardness has been stripped off and therefore they can respond to that over the phone.

HCP 10; geriatrician

Expected practical barriers were sometimes a reality for all 3 groups (with regional variation in health care infrastructure), for example, poor quality connections or lack of digital device; however, they rarely prevented consultations. Over time, familiarity increased confidence, individuals overcame *some* reservations, and some reported improved quality of interactions:

It [telephone consultation] is quite different, but I think I’ve got used to it.

Person with Parkinson disease 13

#### Expected Advantages

Some advantages of remote consultations over in-person consultations, particularly for people with Parkinson disease and caregivers, were reported across the participant groups as having been a reality, including comfort (“Sitting here, he was relaxed” [caregiver 4]) and saving travel (“It did save us a long train journey” [caregiver 11]).

Expectations of improved efficiency and cost-effectiveness existed from participants (“I thought I would be quicker*”* [HCP 19; neurologist]), organizations (*“*Our practice thought that telephone consultations would be quicker” [HCP 13; GP]), and those in authority (“The government and stuff think this is going to save time” [HCP 11; neurologist]). However, HCPs were disappointed to find this was not the case, as more time was needed to circumvent limitations:

At times they’re even taking a little bit longer because you haven’t got your eyes on the patient and you can’t reassure yourself that they look OK.

HCP 13; GP

In contrast, many people with Parkinson disease and caregivers still held this perception that HCPs were “freed up” by remote consultations:

And the doctor is quite busy anyway and I know with a phone call, it frees his time up a bit more.

Caregiver 3

Advantages of video consultations over telephone consultations were frequently described, such as the addition of visual information. Several people with Parkinson disease and caregivers who commented on telephone consultations felt that communication and rapport would improve with video. Some HCP participants with great expertise with video calls reported that with well positioned cameras, body language could be discerned and observational components of clinical examination could be conducted. It appeared that more specialist HCPs (neurologists, Parkinson disease nurse specialists, and neurotherapists) placed greater value in these advantages than generalists (GPs and geriatricians), who were less convinced that the benefits outweighed the obstacles:

I’m not getting that much extra information from a phone call to a video, generally.

HCP 8; GP

Although better than telephone consultation, many participants still felt that communication, rapport, observation, and examination over video consultations were inferior to those in in-person consultations. Subtleties may be lost, eye contact was not possible, field of view was incomplete, and breakdown in digital connection was disruptive.

### Optimizing For the Future

Participants from all 3 groups anticipated that remote technology will continue to be used in health care beyond the pandemic and reflected on how that could best be navigated. Their suggestions cover three domains: “support for people with Parkinson disease and cognitive impairment and caregivers,” “professional development,” and “service improvement.”

#### Support for People With Parkinson Disease and Cognitive Impairment and Caregivers

Given the range of potential barriers to remote consultations, participants felt that support needs should be tailored to the individual user:

Identifying why that person’s a bit afraid of doing that, or put off by it, and then working with that.

HCP 7; mental health nurse

Participants described ways that practical help could be or had been beneficial, with greater need for help with video consultation than telephone consultation. For some people with Parkinson disease, support was required to initiate the call (video or telephone), then it could be undertaken independently; for others, technological checks or a trial run was helpful; and for many participants, troubleshooting technological issues was the priority. Some participants felt that technical training would be helpful, although capacity to learn may vary, and many felt this required a person to teach step-by-step:

It would be very nice if you could afford to have somebody in to teach you how to use things, to make it easier for yourself.

Person with Parkinson disease 9

Actions that people with Parkinson disease and caregivers could undertake to optimize the consultation were proposed, including practicing the technology and reflecting on their condition in advance:

Because you’ve got to be prepared. I did my research, I interviewed my mother beforehand, found out how she was feeling therefore what I wanted to know. So, I was ready for the call.

Caregiver 10

Ways for HCPs to support people with Parkinson disease and caregivers were raised. It was universally emphasized that they required time—to tackle communication barriers, provide explanations and reassurance, and allow for technological obstacles. Several HCPs described introducing the consultation with an explanation of the process and backup plan to reassure people with Parkinson disease:

I explain that all [back-up plans etc] but it’s to reduce that anxiety, and I don’t need to do that when I’m face-to-face, so that’s taking up another ten minutes of my time.

HCP 18; neuropsychologist

Participants from across the groups felt that guidance was needed to set up optimally for video consultations, including camera position and choice of device (HCPs generally recommended laptops over telephones). HCPs described ways to maintain people with Parkinson disease–caregiver balance, such as agreeing a time for the caregiver to leave and ensuring both can be seen on video:

If you set up on a sofa with the iPhone pressed up against your face, which is what people often do, then that isn’t very helpful really. Whereas if you were to have it on a table with a couple of chairs behind it so that you’re getting a good view of the person, a good view of the relative, you can interact with both of them, and you can have some room behind them to get them to walk.

HCP 11; neurologist

Many participants described the existing instructions provided for using technology, but also felt that it needed simplification, and in some cases, written information was not sufficient:

Some of the information that is provided to help you solve problems that come along is not as clear as it might be...Partly language and partly generations I think. People who live in certain environments, in IT environments, learn to have their own language and think everyone else understands it.

Person with Parkinson disease 1

#### Professional Development

HCPs held varied views about training for remote consultations. Some felt that attitudes toward video consultation needed to change first, through better understanding of the benefits. Many participants identified an initial hurdle that required optimism and confidence to jump. HCPs recalled experiencing or witnessing improvement and increased confidence over time—a participant recalled having previously found video consultations “much harder” and “come out feeling quite tired” (HCP 11; neurologist), but this had improved:

I think a lot of it is just being familiar with what you’re doing, being happy with using the technology and using your devices and so forth.

HCP 11; neurologist

Varying degrees of confidence in using technology were expressed. Some had received training on the digital platforms, many had picked it up through use, and others felt they needed training to get started. Similar to people with Parkinson disease, many HCPs desired ongoing support and troubleshooting rather than training*.* Although generally feeling confident using video technology themselves, several HCPs felt that they could not help patients if something went wrong at their end.

Beyond technology, some HCPs felt that remote consultations required the same skills as in-person consultations, whereas others felt that they demanded new trainable skills. Some of the techniques used for in-person consultations were described to be adapted for telephone and video consultations:

The same as phone consults; trying to build that rapport, the active listening skills, and you just need to be a little bit more pronounced in your active listening.

HCP 13; GP

Some HCPs described modifications to their consultations; for example, questions to remotely assess cognition or subjective reports of function where objective physical measures would have been used in person, but several participants desired a standardized approach:

What I would like: a validated video exam that we all get used to doing. It’d be nice to get a validated telephone exam.

HCP 14; neurologist

There was a sense among many HCPs that what an *optimal* remote consultation entailed remained unknown; several participants asked what other participants had said or described learning from colleagues. All participants, especially HCPs, shared recommendations for HCPs undertaking remote consultations, as summarized in Multimedia Appendix 2.

#### Service Improvement

Most participants across the groups favored a blended model for the future—in-person or remote consultations depending on context, necessitating changes to services to enable personalization. Participants described who remote consultations should be used for, how services need to adapt, what is needed to deliver a better service, and why improvement is needed.

##### Who?

Remote consultations were felt to be most suited for routine appointments for stable conditions and when a person with Parkinson disease.

HCP relationship already existed, whereas in-person consultations were thought to be better for complex cases or those experiencing complications and consultations involving significant discussions (eg, advanced care planning):

The only time you need to see a doctor, I think, if things are not going too well.

Caregiver 3

However, caution may be needed. Some people with Parkinson disease hypothesized that if their appointment were changed to in-person consultation, they would anticipate bad news:

Trouble is if the doctor says to you now, “come in and let’s talk about it” then you start to worry even more.

Person with Parkinson disease 5

Overall, participants felt that the method of consultation should be tailored to the individual by assessing the pros and cons on a case-by-case basis; by considering the resource, access, and capability of the individual to use remote communication technology, in particular, considering their communication and cognitive symptoms, to ensure that value is added to their care; and based on the preferences of people with Parkinson disease and caregivers:

...For lots of things, it has been useful. And then for certain people, it’s just not useful at all. So, it is again about thinking about the individual and what is potentially best for them.

HCP 5; OT

##### How?

Participants discussed how this can be operationalized, potentially using telephone triage and categorizing to consultation type. Several participants emphasized the importance of contingency planning, for example, being able to undertake in-person assessment if the remote consultation is unsuccessful.

For service delivery, all participants felt that having flexibility and adequate time was essential, with many HCPs emphasizing that remote consultations did not save time. Sometimes separate consultations for people with Parkinson disease and caregiver may be required, and some participants felt that more frequent appointments were preferable over very long ones to reduce the risk of tiring. Some HCPs had experienced problems of fixed scheduling, whereas others positively recounted flexible systems:

The nice thing about telephone consultation clinics is actually there’s a bit more flexibility so we’re not giving patients specific times of when they’ll be called, we’re giving them windows. So we can be slightly flexible if people then say, “No, can you call me at this time?”

HCP 8; GP

##### What?

There was evidence of variation in equipment availability, administrative support, and suitable environments across services, which correlated to the apparent success of remote consultations. Use of asynchronous remote communication, such as simple and responsive SMS text messaging and emails were valuable for some participants from all groups. Overall, the need for improvement to infrastructure was emphasized:

...Just making sure every computer you use has got the access to it all, I think that’s really important.

HCP 25; OT; Parkinson disease service

Several participants across the groups reported issues related to people with Parkinson disease lacking simple and suitable devices for video calls. Many HCPs felt that the platforms currently used in health care settings needed to be improved. Many participants reflected that platforms popular for personal use, such as Zoom, Skype, and WhatsApp, were more easily managed and that familiarity could help in overcoming barriers:

People that had previously been a little bit, “oh, I’m not sure about the technology,” realized they were quite capable of using Zoom, it was an easy platform.

HCP 20; speech and language therapist

##### Why?

The importance of improving services was emphasized by several participants, typically citing concerns about exclusion through “provision disparity” (HCP 8; GP) or competence and confidence in using them:

The people that do take the service up are probably the people that least need it.

HCP 12; Parkinson’s UK advisor

## Discussion

### Summary

HCPs, people with Parkinson disease, and caregivers perceived remote interactions as more transactional, lacking personalization, and challenging for building rapport. They questioned whether remote consultations could be used as a substitute for *real* in-person consultations. Limitations of remote consultations were perceived, particularly, in conferring great risk. These issues were more prominently perceived for telephone consultation than video consultations, but existed for both modes of communication, with most participants considering them inferior to in-person consultations.

Issues for this population were intensified through remote technology, including communication and cognitive challenges, balancing people with Parkinson disease and caregivers within consultations, and significant discussions (eg, about the future). Perspectives had evolved, with some anticipated barriers not materializing (such as age being a restriction to access) and some expected advantages not coming to fruition (such as saving time). Although participants were generally surprised by the relative success of remote consultations and confidence in remote technologies was increasing, most participants still preferred in-person consultations. People with Parkinson disease, and caregivers, compared with HCPs had divergent perceptions about efficiency of remote consultations, with the former reporting them to improve efficiency and save time, but the latter typically rejected the notion of time being saved. Participants proposed ideas to improve services, anticipating a combination of remote and in-person health care consultations moving forward.

### Context of Existing Literature

To the best of our knowledge, this is the first study to explore remote consultations for people with Parkinson disease in a real-life setting, to explore these 3 groups’ perspectives, and to focus on people with Parkinson disease and cognitive impairment.

Both human and technical aspects of telemedicine have been identified as contributing to quality [32], which were also apparent in our study. Within Parkinson disease, telemedicine has been shown to be both feasible [11-14] and associated with high rates of satisfaction both in studies [13,14,18,33,34] and in the limited reports of real-life application [35,36]. Studies have been small and heterogeneous (eg, regarding frequency of consultation and whether telemedicine replaced or supplemented routine care) and produced mixed results regarding quality of life and clinical outcomes [37]. As such, effectiveness of remote models compared with in-person consultation remains inconclusive. The advantage of reduced travel burden for patients and the barriers from technological problems and limited physical examination have been consistently reported. Studies have recruited predominantly digitally literate, well-educated, White samples, which may not be representative of the wider population with Parkinson disease [37], and few studies report cognitive status. Studies typically provided equipment, software, and technical support, with consultations delivered by clinicians trained and experienced in telemedicine, which may not be applicable to standard clinical care models. This study gives insight into the real-life experiences of clinical remote consultations in a typically understudied population, within the UK National Health Service. An evolving body of literature, typically based on HCP reports of personal experience, offers tips to clinicians undertaking remote consultations [38-41]. This study bolsters this with the patient and caregiver perspective and nuance for this population.

Accounts of remote consultations as “transactional” are consistent with those reported in the study of other conditions and contexts [4,42]. An analysis of primary care telephone encounters found more biomedical information exchange than psychosocial communication, and the telephone consultations were a less patient-centered approach, which could be attributed to the short duration of interaction [42]; however, in our study, remote consultations were not thought to be shorter in this population. The relationship between duration and quality of consultation is debated [43,44]. Participants in our study strongly believed that more consultation time was beneficial, perhaps reflecting the condition complexity. However, the inconsistency suggests that loss of personability remotely is not purely time driven. A qualitative study of neurology consultations identified a “business-like” style and ability to “take control” in remote consultations, which were perceived as advantageous. However, the perception of the dynamic as “transactional” was portrayed as a *disadvantage* by people with Parkinson disease, caregivers, and some HCPs in our study. The reduced HCP enjoyment of interactions when remote resonates with reduced consultation satisfaction previously reported [4].

Although not widely reported previously, the perception of remote consultations as not being *real* resonates with a primary care study reporting that some people expected telephone encounters to determine if or when they would be seen in person [42]. This may be more pronounced in this study owing to the rapid shift to remote consultations during the pandemic and highlights the need to promote understanding of their purpose. The perception of increased risk with remote consultations is mirrored in studies of clinicians’ perspectives within primary and secondary care [4,45]. The importance of observation and physical examination is particularly widely reported in neurology [4] and Parkinson disease [6,16,18]; however, there has been less attention to clinicians *sensing* clinical judgments, which was marked in our study. Clinicians’ *sixth sense* has been discussed in psychology and acute care patient safety literature [46], but perhaps is more widely applicable.

Communication problems in Parkinson disease are well known [47,48], and health communication research has long established the importance of nonverbal communication [49], which is unavailable in telephone consultations. Difficulties relating to memory and discussion being directed to caregivers with risk of exclusion of the patient themselves have been reported in remote consultations for dementia [20]. Cognitive impairments are widely perceived to be potential barriers to remote consultations [4,39,40,50]; consideration of mental capacity for suitability of remote consultation is highlighted in the UK General Medical Council guidance [51]. The effect of nonmemory cognitive impairments, such as executive dysfunction [52], alongside speech and behavioral symptoms, may create even more difficulty in sustaining complex discussions for people with Parkinson disease. This is particularly relevant for significant discussions (such as diagnoses and prognoses), which are difficult remotely, across disciplines [4,53].

In a recent study of remote primary care consultations for people with dementia [20], older age conferred more barriers, but this was not replicated in this study, where mixed experiences were reported, but not predictable from age. Instead, familiarity with technology was a facilitator; however, those with more significant cognitive impairment may have lost digital skills and confidence or be unable to transfer it to a new context. Increased confidence with remote consultations over time has been recognized during the pandemic [45,54], thus supporting the concept of practice. Advantages regarding convenience and comfort for remote consultations appear widespread [4,6,53], but perhaps more so in Parkinson disease owing to exacerbation of symptoms with stress [55,56]. A qualitative study of the effects of the COVID-19 pandemic, which touched on remote consultations [57], and another study of experiences of people with Parkinson disease and HCPs regarding Parkinson disease management during the COVID-19 pandemic [19] similarly found mixed opinions of remote consultations. In the latter, several HCPs reported improved service efficiency, which was not experienced by the HCP participants of our study. This may be a particular issue for those with cognitive impairment, which was not explored as a factor in either of these studies.

The need for evolution of platforms, infrastructure, and resource within clinical health care systems such as the National Health Service, while preventing health inequalities, corresponds with other UK-based studies of remote consultations [4,53,54], but with specific needs of this population: time, simplicity, and flexibility. Flexibility is recognized to be necessary in delivering personalized care [58]. The expectations of remote consultations are varied, and importantly, perceptions of efficiency and saving of time differed among people with Parkinson disease and caregivers, compared with HCPs. This discrepancy may lead to dissatisfaction on both sides. Our findings highlight that *cognitive impairment* covers a range of abilities and support for individuals varies; therefore, blanket procedures will not be appropriate. The barriers to remote consultations were mostly portrayed as *challenges* rather than absolute *disadvantages*, perhaps owing to the expectation that remote models of care will continue and the hope that these issues can be surmounted.

### Strengths and Limitations

This is the first study including an underrepresented population (people with Parkinson disease and cognitive impairment) and triangulating the perspectives of patients, caregivers, and HCPs. Conducting the study remotely enabled inclusion of health services from multiple geographical areas, and snowballing enabled a wide reach, but may have predominantly reached individuals with specific interest in the topic. Inclusion of participants with subjective cognitive symptoms rather than a formal diagnostic process prevented being restricted by underdiagnosis, which is a recognized problem [27]. However, we cannot formally consider interpretation by objective severity of impairment. As has been a long-standing issue in Parkinson disease research [59], challenges were faced in recruitment of ethnic minority participants, despite targeted efforts, which may limit the applicability of the findings to these groups. Clinical audit data show 92% of people with Parkinson disease in neurology and Elderly Care Services in the United Kingdom to be White individuals [60], but even the use of primary care recruitment in ethnically diverse areas did not increase the diversity of our participants. Validity of interpretation was ensured through PPI consultation and a multidisciplinary clinical and academic team.

An unavoidable challenge of research in this population is that the condition often causes communication difficulties. Some participants had difficulty in expressing their views, and caregivers proxy views could be biased. Individuals who are not comfortable or able to communicate via telephone or video or with limited English language skills may be underrepresented. Although the range of professional backgrounds represented brings richness to these data, it is important to recognize regional variation in health services [61]; many people with Parkinson disease will not routinely encounter this range of specialist professionals [62]. The study was conducted within the United Kingdom and may not be representative of health services in other countries.

### Implications for Clinical Practice and Research

This study adds to the literature on remote consultations, with consideration to this subset of patients and caregivers. Although it was clear that care and consultation method needs to be personalized to the individual, awareness of these issues and the suggested improvements can help to manage expectations and optimize remote interactions, as summarized in Textbox 3. Future studies should continue to evaluate remote service delivery in real life as it evolves and as the pandemic situation changes. Further studies on advantages of video consultations over telephone consultations and on asynchronous remote e-consultations with people with Parkinson disease would also be valuable.

**Key messages for clinical practice.**

**Lessons for health care professionals (HCPs)**

- HCPs should be aware of the perceived transactional nature of checklists and closed questions.
- HCPs should be aware of potential exclusion of the voice of people with Parkinson disease.
- Pauses by telephone can be difficult to interpret, but caution must be taken to not interrupt as they may need more time for communication.
- Manage expectations, clarify the role of the consultation, and offer reassurance and a backup plan.

**Tips for people with Parkinson disease and caregivers**

- Practice using the technology and platform in advance.
- Preparation can improve the quality of consultation:
  Reflect and record points for discussion in advance.
  Optimize the environment and device used for the consultation.
- Inform the health care providers about the better times for your condition, eg, when medication is working best.

**Considerations for service design**

- Written guidance for remote consultations may not be sufficient to enable use. Guided use of technology may be necessary for people with Parkinson disease and cognitive impairment and caregivers.
- Services should be flexible, enabling individually optimized timing and communication methods for interactions and avoiding exclusion of those with impairments that affect use of remote interactions.
- Platforms for remote consultations should be simplified by using familiar concepts from those widely used for personal communications.
- Telemedicine should not be assumed to be quick or more efficient—more time is needed for consultations with this population; however, this may be best achieved through increased frequency of appointments to minimize risk of tiring in very long appointments.

### Conclusions

Many advantages and challenges of remote consultations are universal, but there are some specific issues to consider for those with cognitive impairment in Parkinson disease, owing to the combination of physical and cognitive symptoms and psychological factors, such as exacerbation of impairments because of anxiety. HCPs, people with Parkinson disease, and caregivers perceived remote interactions as more transactional, lacking personalization, challenging for building rapport, not *real* consultations, and riskier owing to their limitations. This applied particularly to telephone consultations, but also to video calls, to a lesser extent. Access and technical barriers limited the use of video consultations. In contrast to perceptions of people with Parkinson disease and caregivers and reports in previous studies of people with Parkinson disease, HCPs denied time being saved with the change to remote consultations.

Although challenges and descriptions of negative experiences were universal, in practice, remote consultations had worked better than expected by many participants, and some anticipated barriers were not actually experienced; for example, many older people were unexpectedly accessing consultations remotely. These experiences should be considered when planning future remote health care for people with Parkinson disease.

## Appendix

Multimedia Appendix 1 Additional quotes from participants.

Multimedia Appendix 2 Recommendations for health care professionals undertaking remote consultations.

## Abbreviations

GP: general practitioner
HCP: health care professional
NIHR: National Institute for Health Research
OT: occupational therapist
PPI: patient and public involvement
