# Supplementary material for: Remote Consultations for People With Parkinson Disease and Cognitive Impairment: Interview Study With Patients, Caregivers, and Health Care Professionals
Source: JMIR Neurotechnol. 2022 Dec 2;1(1):e39974. doi: 10.2196/39974 (PMC12671285; doi:10.2196/39974)
Supplement: Multimedia Appendix 1 [file neuro_v1i1e39974_app1.docx]

**Theme 1: The Nature of Remote Interactions**

| **Subtheme** | **Additional Sample Quotes** | | |
| --- | --- | --- | --- |
|  | **People with Parkinson disease** | **Caregivers** | **Health care professionals (HCP)** |
| **A Transactional Exchange** | “I wasn’t that impressed with, it was just a whole series of generalised questions rather than focused on me and on what I’ve been saying and on my this or that. Um and the good clinician I would’ve thought can very quickly pick up what the key issues are, and focus on them and get a conversation going… But sometimes you feel things are a bit on the automatic side” Person with Parkinson disease 1  “We went through a list” Person with Parkinson disease 13 | “…the technology kept crashing every 5 minutes so we didn’t get the full time, and she had to give me extra sessions to make up for it. And I think it detracted from the continuity. So, we’d end up sort of… it’s always talking, and then laughing about the technology. And that broke up the flow of what I needed.” Caregiver12  “… sometimes the reason you want to talk to the doctor is just that you’re a bit worried about whether you should be worried. And it’s not necessarily that you could say ‘this thing is wrong’ it’s a bit nebulous. I think, maybe it’s harder to convey that on the phone.” Caregiver2 | “I’m using a lot more closed questioning at the end in terms of specific questions when people are presenting with a new complaint, being very direct, still open but really having to tease out the history and probably being more directed because you're not then able to learn as much from a clinical examination obviously.” HCP8 (GP)  “And, now my clinic note is automatically set up so I know I’m going to go through that list, so I think that helps, otherwise I just get stuck, so that works for me.” HCP19 (Neurologist) |
| **Is it “Real”?** | “…doing it all from arm’s length … It’s been a bit frustrating. Because I wasn’t, if I’m being honest, I wasn’t totally sure that they were being that straight with me.” Person with Parkinson disease 1  “there are times when I think I’m not sure what the voice therapist is going to do… It’s like looking down your throat on the phone. It’s not possible.” Person with Parkinson disease 13 | “I don’t know how – they could see her fully; they’ll be able to see her face.” Caregiver15  “I think it was this idea that you needed to see a patient that I’ve always been brought up with. You need to palpate a patient, you know, and I think that’s still true in many ways... So actually I suspect this is an old-fashioned age thing that I do think having a physical relationship, the initial meetings, and maybe a few and then... I just wonder if that makes the difference.” Caregiver11 | “Some of them don’t like it at all, they don’t like it, they don’t see it as a consultation, they don’t see it as achieving anything.” HCP22 (PDNS)  “I think initially people were like, “If you're not seeing me it doesn't count.”” HCP8 (GP)  “I always get the impression, and maybe this is me just projecting, that they're seeing the phone call as a step to being seen” HCP13 (GP)  “they’re just quite happy to have a chitty chat on the phone.” HCP23 (Geriatrician) |
| **A Risky Process** | “I think neurologists in particular get a lot of information visually, you know the moment they see you – they see you walking, doing this doing that doing this. They can diagnose you before you’ve sat down. So it’s quite difficult them not having sight of you.” Person with Parkinson disease 1  “But it’s difficult not to make – come to conclusions which are based upon missing – which are missing some of the very practical assessment” Person with Parkinson disease 13 | “Because sometimes I think they can see how she’s progressed or – by seeing her. And my mum’s got a habit of going, “No, everything’s OK; yes, that doesn’t bother me,” I suppose, over the phone [laughs]. And if they could see her, and know she’s seeing them, then she’s a bit more honest, I think.” Caregiver15  “I think the risk is that you could have a Parkinson’s patient on a telephone call or a video call where they couldn't have privacy. Whereas if they walk into a room and the door is shut” Caregiver11 | “… I’m sat in an open plan office with lots of people behind me.” HCP22 (PDNS)  “On the phone you can ask all the questions, you can ask exactly the same question, but seeing, it just gives you that extra layer of reassurance, I feel.” HCP15 (GP)  “If the patient is on their own… And he’s [PwP] sitting on a bar stool in the kitchen and I’m thinking, “I don’t dare ask you to stand up.” Because I really don’t know what’s going to – it’s not safe.” HCP27 Physiotherapist)  “I mean honestly I do worry, I worry it’s definitely not adequate… So there are a lot of concerns there about whether the things are being translated or done properly” HCP14 (Neurologist) |

**Theme 2: Challenges Exacerbated by Being Remote**

| **Subtheme** | **Additional Sample Quotes** | | |
| --- | --- | --- | --- |
|  | **People with Parkinson disease** | **Caregivers** | **Health care professionals (HCP)** |
| **Communication & Understanding** | “It [telephone calls with more than 2 people] starts to become more difficult, more confusing because you don’t know who you’re talking to…” Person with Parkinson disease 5  “[using technology-] I find it very hard… to remember, that’s what’s happened.” Person with Parkinson disease 9  Regarding video calls: “Well, I do feel a bit remote… by the time I get my answer out in the open, I’m answering a different question.” Person with Parkinson disease 11  “I always end up in a dead end, I lost my way I think on the computer or i-pad. It just never seems to work for me.” Person with Parkinson disease 4 | “he finds it very difficult because his speech, it’s very soft and he can’t find the words that he wants to say. And it takes him a very, very long time… He feels awkward and uncomfortable on the phone… and he gets quite upset because he can’t deal with it.” Caregiver12  “When we’re together, whenever that last was, they [family] will wait for him, whereas electronically that’s more difficult.” Caregiver11  “My father might not even remember what his symptoms have been prior to a phone call with the doctor.” Caregiver2 | “…the speech was so challenging that it was just very, very difficult to get any kind of meaningful content.” HCP21 (Psychiatrist)  “But if the patient is living on their own it doesn’t work, they haven't a clue sometimes what you’re calling them for, they can’t remember why they called you, they’ve got no idea what medications they’re on, nothing.” HCP26 (PDNS)  “I have tended to find that people can sit back a bit on a video call, rather than engaging and being present… some people with cognitive changes are a little bit more stepped back.” HCP20 (SLT) |
| **Interpersonal Dynamics** | “Yeah I was sitting with [name of caregiver] which we do most of the time, and I just thought if I misunderstood a question or whatever, [name of carer] will have a go and try and explain it to me and I can then answer it.” Person with Parkinson disease 3  “How do you feel about me being with you [for the telephone consultation]?” Caregiver11 … “Oh yes, I have to have you there.” Person with Parkinson disease 11  “Well, quite honestly, [Caregiver4] does most of them [video calls], but I don’t do it very much, very truly, very unusual, on the computer or i-pad.” Person with Parkinson disease 4 | “If I am with him on a zoom call, he tends to be completely silent. He just sort of cedes the speaking role completely. And I assume it, you know, just for the sake of time I suppose. But I think he understands what’s going on…” Caregiver2  “So it’s hard… it’s always me, I’ll call the doctor...” Caregiver15  “He has to have me there. Is that because I made [laughter] the phone call or I insist?” Caregiver11 | “I try and talk to the patient but you will spend the majority of the consultation with the other half, so you’re more reliant on what they’re saying. And, again, if you’re doing a video and they’ve got a cognitive inability, it tends to be their other half and they might just be there in the room but they’re not contributing.” HCP26 (PDNS)  “It’s hard sometimes when a family member takes over the conversation because you can’t – whereas in clinic you can say ‘no’ and make it quite obvious without being so blunt…” HCP8 (GP)  “So I haven't found a satisfactory way other than, as much as I hate to admit it, side-lining the patient and talking to carer and making decisions with the carer.” HCP14 (Neurologist) |
| **Significant Discussions** | “I think, because that [diagnosis] obviously is a major moment in anybody’s life, so it was probably better to be given that diagnosis face-to-face.” Person with Parkinson disease 8  “Maybe, maybe if it was something serious, or what I thought was serious then maybe I might not be quite so happy [with telephone consultation]” Person with Parkinson disease 5 | “Yeah, um, I think not so great. I mean you know, it’s hard enough in person. I mean, yes. But you know, ideally, ideally you would want to sit down with, you know, with somebody.” Caregiver2  “It’s obviously better to have it [discussion about the future] all on a personal basis… if there is any bad news or something, one doesn’t like to hear it on the phone.” Caregiver7 | “I don’t think diagnosis should … well, not big diagnoses, should be given over the phone, I think that’s something that people need time to digest, and you need to look at the body language and gauge what you need to say or what you need to do support.” HCP26 (PDNS)  “Talking about the future and talking about the progression, it’s very difficult to have these conversations either over the phone or through the technology, even seeing the patient” HCP4 (PDNS) |

**Theme 3: Expectation Vs Reality**

| **Subtheme** | **Additional Sample Quotes** | | |
| --- | --- | --- | --- |
|  | **People with Parkinson disease** | **Caregivers** | **Health care professionals (HCP)** |
| **Anticipated Barriers** | Regarding video consultations: “I think I could be quite open with them” Person with Parkinson disease 9  “The zoom is something new. I wouldn’t normally have done that. It’s also a generational thing, you know I’m not that comfortable with all that IT stuff. It’s a bit of a pain…  And you can quickly get quite frustrated and quite lost [online]. I mean it’s common... If you ask me what is the most frustrating thing in my life, one of them is the computer.” Person with Parkinson disease 1  “Yeah it was ok, well I mean, you know, these days you get used to like you, like you’re doing it now over Zoom.” Person with Parkinson disease 5 | “I think that’s only us two who find it [video calls] fiddly. I think the others [family], who use technology all the time and they're doing Zoom meetings at the moment at work and this sort of thing, are completely unfazed by it, if I’m truthful.” Caregiver11  “My husband can’t really use the phone; doesn’t really understand how to move, which bits of screen used which you’d use your finger on… Technology is great if you have the mental capacity to deal with it.” Caregiver12  “Because my computer is so old it hasn’t got the old cameras on the top or anything it’s just a basic computer.” Caregiver3  “in areas like we are, as a lot of places, our broadband can be shocking.” Caregiver11 | “initially when I was told I was doing phone clinic I was very dismayed, but actually I haven't found them difficult… I assumed, I was thinking, we’re doctors, this is our art, how can we possibly not examine our patient and look at our patient. And I still agree with that to an extent... But I feel I get enough, and you focus so much more on the history, which we all know is the key in any consultation on the patient’s account, so, yes, it’s been a nice surprise.” HCP10 (Geriatrician)  “But some of them manage it really well, you know, you’re amazed thinking, oh, my god, how old are you, you know, and they’re absolutely there on it, sorting it out, they do.” HCP26 (PDNS)  “But to be fair, most of the clients that I’m dealing with, some of them haven't even got a smartphone, so it does make it quite difficult really.” HCP12 (PUK Advisor) |
| **Expected Advantages** | “I think you haven’t got the journey to have to go there, for a start. And the second, then … you’re going to do the same as if when you see … you know, if I came to see you as the doctor, I’ve got even more of your time, because you’re actually talking to me. When you go to appointments, you can have so little time, because they’ve got the next one waiting.” Person with Parkinson disease 9  “The interaction [by telephone] between me and the doctor was quite… It was quite- more relaxed. I think I was more relaxed” Person with Parkinson disease 4  For in-person consultations: “I don’t mind the cabs, but the money...” Person with Parkinson disease 11 | “It’s [remote consultation] much better, and it frees them up more, as well, I think.” Caregiver4  “it’s [remote consultation] fine, because going to [name of hospital] isn’t a pleasant journey!” Caregiver3  “I haven’t actually done it [video call] to any professionals. That’s only been to family. And you know, that’s fine, because even if you cut out or you’re talking as though you’re under the sea, that’s – it’s fine. It’s doable. It’s just that, when you’re talking to a professional or you need to get, like, important information across, then that becomes an issue.” Caregiver14  “In a way it’s more convenient. Doesn’t take as much time. On the other hand, you, you know if you’re told you’ll get a call back in the afternoon, it means you’re sort of waiting for it the whole afternoon.” Caregiver2 | “it’s better that I thought it was going to be when I started using it, but not the panacea that some people think it is.” HCP18 (Psychologist)  “I had plans at the beginning I thought, I can do two clinics in an afternoon. It’s not quicker, sometimes it’s longer and sometimes patients are quite lonely and they spend a long time chatting, so sometimes it’s longer, but it’s about the same.” HCP19 (Neurologist)  “…people like the government and stuff think this is going to save time. It is not going to save time. OK? It is just a different way of doing things, and it saves patient travel and it may improve patient experience, and it potentially may even improve some clinician experience. But it is not going to improve efficiency or save time.” HCP11 (Neurologist) |

**Theme 4: Optimising for the Future**

| **Subtheme** | **Additional Sample Quotes** | | |
| --- | --- | --- | --- |
|  | **People with Parkinson disease** | **Caregivers** | **Health care professionals (HCP)** |
| **Support for People with Parkinson’s and Cognitive Impairment & Caregivers** | “Yeah, sometimes I let that happen [someone else dial], because sometime I can’t get the numbers quick enough, and I keep cutting – keep going to the wrong places so I have to get the children to … They’re dialling, and I could talk to him then.” Person with Parkinson disease 15  For HCPs conducting remote consultations: “just be natural, yourself. Which you can be. Because sometimes you’re, like, scared, when you haven’t seen somebody for a long time and you’re frightened to – I just find it’s more approachable… And that people would think that it’s more relaxing for them.” Person with Parkinson disease 9  “Yes, normally before I go to an appointment or have any appointment I write a note, which is a reflection of any developments in my symptoms, a reflection on anything to do with the medication and any other particular issues or comments I have to raise. I – I try and do that because I think it kicks it off.” Person with Parkinson disease 1 | “So maybe just to be more patient, to be – to be able to listen, to make sure it’s a good line. Yeah, that’s very important. If they’re comfortable and they’re in the mood to talk at that time. Because things could be going on around, as well… So that’s, I think, should be taken into consideration… I suppose patience… “Do you understand everything I’ve said to you? Do you need me to explain the question, or to explain?” or, “is there anything you need to say?”, always iterate that… “Don’t be afraid to ask; it’s not a bother.” Because sometimes I think people think it’s a bother to ask doctors and to ask people things.” Carer15  “They still have to have the patience though [remote or in-person], don’t they? And it’s the patience that’s the problem.” Caregiver11  How to improve telephone consultation: “I suppose if there was just some room for talking about um, how, whether, well how worried I am or, or the acknowledging that this could just be a sort of, um, sort of exploration of a possible problem rather than – I don’t know.” Caregiver2 | “…somebody with cognitive impairment they might have difficulty following certain things, you know, in order to log in. That’s why I think sometimes it’s best to call them, and also, don’t worry, if you can’t log in I’m going to ring you, just don’t worry. There is a lot of anxiety.” HCP16 (PDNS)  “Practical stuff, you know, sometimes getting people to set the camera up… But, prior to that someone else has already checked that they’re at the right time, they’re expecting to see the right person, almost like a reception at a hospital. And, that their sound is working, their video is working, and if they’re not then they go through a few check throughs with that. So, I don’t tend to do that with people, but I think checking on that, even having a trial run, or just having the first one as just about using the tech and build it up very gradually and slowly.” HCP9 (Psychologist)  “being there when they do it just so that they know that if something’s gone wrong, then someone there is going to be able to rectify it for them. So just little things like that really would help enormously, I think.” HCP12 (PUK Advisor)  “The Parkinson’s support group that they’re part of, they have regular Zoom meetings… And the person from the support group was going round and helping people to set it up, if they hadn’t already set it up.” HCP6 (SLT) |
| **Professional Development** | “some proper training for staff… to ask the right kind of questions actually. They can’t see it. They can’t see you, you know an awful lot. How are they going to replace that when they’re on the phone? What kind of questions are they going to have to ask? What kind of conversations are they going to have to try and get some of that information…there should be advice, and there probably is, to doctors and people about how they should deal with these things. What they can get out of some conversations and what they can’t. When they need to see patients, kind of thing.” Person with Parkinson disease 1 |  | “Try it out. Yes, it’s just getting over trying it out. We are naturally procrastinators, and if it looks hard we will have difficulty starting off doing it.” HCP11 (Neurologist)  “I think an allotted almost training block or day, where you’re learning how to use this new bit of software and you can navigate around it, therefore, you’re better at helping other people navigating around it, yes.” HCP9 (Psychologist)  “Yes, I think we need, I guess different skills for telephone consultations, different skills for video.” HCP14 (Neurologist) |
| **Service Improvement** | “If, if it’s a routine follow-up then I’m, I’m happy either way. I think if it’s, if it was bad news I’d rather be there in person… Trouble is if the doctor says to you now, ‘come in and let’s talk about it’ then you start to worry even more.” Person with Parkinson disease 5  “if you haven’t changed too much, then I think it would be, yes. I think it [telephone Parkinson’s appointments] could work.” Person with Parkinson disease 9  “Yes I think one thing would be to get a group of users into a room, with some people who are creating the programmes. Just in the Parkinson’s area, for example, if there are things which were being used and occasionally which broke down – to make sure that people could very easily solve it or one way is to get a group of people together to talk about it and to say – what have been your problems with IT? and what do you find difficult?” Person with Parkinson disease 1  “And I can’t imagine, with Parkinson’s, not seeing people. Well I am saying that about clinicians. With things like you’re suggesting [self-management support], which is how you should do this or that, you can get a lot out of just talking. You don’t need to see people if you’re talking about managing your situation or what sorts of exercises are good – I mean exercises yes, but as a generalist view.” Person with Parkinson disease 1 | “Going back to when we were saying about support for carers, the phone call; I think that’s different, that’s making contact in some way. But for something that needs to be longer and a bit like this. I’m finding this much better than just on the phone.” Carer12  “You know I think, you know, it’s a good idea to have a phone conversation or video call with a doctor on both parts. The only time you need to see a doctor, I think, if things are not going too well then you, you know, you say could I make an appointment and see you at some point because I feel things are not going right. Then they say ok we can do that, set a date for that. What’s the problem and get you to come in.” Caregiver3 | “I think it’s easier on the phone when you actually know somebody… I think when you’re dealing with new patients, you know, this sort of thing, you really do need that face to face and see what the problem really looks like and get to know the person a bit better.” HCP23 (Geriatrician)  “…if it’s someone who’s able to provide a clear history and access exactly what their needs are, or if they have a relative who’s able to describe their needs in detail. If they don’t fit into those two categories, then I don't think there's always a huge value in it [remote consultation].” HCP10 (Geriatrician)  “there’s quite a few people where I would phone them up, do the majority of it over the phone and then say, now I’m going to flip to video… I would use a form of video technology that is very easy for elderly people to quickly use on their phone.” HCP19 (Neurologist)  “It [telephone] doesn’t work when there is complexity… a single problem, focused issue then you can really hammer down and nail it and it’s OK.” HCP24 (Palliative Care Physician)  “if you have any doubt, any doubt at all about what you’ve been told or what you’ve seen, you want a soon follow up face to face appointment.” HCP22 (PDNS)  “I think, because of the platform we’re using, the process is just not friendly for somebody with cognitive problems.” HCP27 (Physiotherapist)  “So I think there's a slight danger in doing video of doing videos for people who are better off, because I think phone videos with patients doesn't work very well. So there's that kind of provision disparity.” HCP8 (GP) |
