# Supplementary material for: Remote Consultations for People With Parkinson Disease and Cognitive Impairment: Interview Study With Patients, Caregivers, and Health Care Professionals
Source: JMIR Neurotechnol. 2022 Dec 2;1(1):e39974. doi: 10.2196/39974 (PMC12671285; doi:10.2196/39974)
Supplement: Multimedia Appendix 2 [file neuro_v1i1e39974_app2.docx]

**Supplement 2.** Tips and techniques obtained from participants from all groups for health care professionals undertaking remote consultations with people with Parkinson disease and cognitive impairment

| **Period and aspects** | **Recommendations** |
| --- | --- |
| **Before the consultation** | |
| **Environment** | - Consider the environment of people with Parkinson disease—comfort, reducing stress, and privacy - Consider the health care environment—privacy, ambient sound, and equipment |
| **Patient-focused preparation** | - As for an in-person consultation, read the relevant clinical information before the consultation - Where possible, determine the preferred method of consultation for that individual and identify how they best communicate and if any support is needed - Consider the best time of day for that person, eg related to their medication times and responses |
| **Prepare for the technology** | - Familiarize and practice with the technology; for video consultation, ensure adequate screen size or 2 screens - For video consultation, consider accessing the platform via patient access to be able to guide them through difficulties |
| **Preparatory instructions and guidance for people with Parkinson disease and caregiver** | - Simplicity is key - Encourage reflection of their condition, by noting any issues or questions they want to be addressed in the consultation - Consider practical advice, eg, camera positioning for video calls - Tailor to the needs of your consultation, eg, device preference—laptop may be preferred over telephone for multiple participants to ensure that all are visible and for examination (eg, to include space for gait observation), whereas a mobile device may be preferred if review of the wide home environment is desired |
| **Practical support for people with Parkinson disease and caregivers** | - Depending on availability within the service and tailoring to the individual needs, consider identifying if there is someone who could help by doing the following: - Checking their technological setup in advance - Providing a practice run - Providing technology training - Initiating the call and then handing over to the person with Parkinson disease - Guiding them through the whole consultation - Being available to troubleshoot in advance and during the consultation |
| **During the consultation** | |
| **General consultation management** | - Explain the process of the consultation at the start, acknowledge the difficulties, and offer reassurance and a “backup plan” in case technology fails - Ask who is present - Minimize distractions - Consider taking notes |
| **Making the best of technology** | - Speak loudly and clearly, looking at the camera - Consider using audio from telephone alongside video for image - Consider using a second screen for clinical records |
| **Communication skills** | - Be confident to reduce their anxiety - Take it slowly, use simple and concise sentences, avoid frequent or abrupt subject changes, and allow opportunity for questions - Be friendly to build rapport - Frequently recap and check understanding to support cognitive impairment - Help when people forget or offer to come back to it |
| **Awareness of the person with Parkinson disease–caregiver dynamic** | - Make a conscious effort to include people with Parkinson disease by speaking to them directly - Consider agreeing at the start about how the time will be split between the person with Parkinson disease and the caregiver |
| **After the consultation** | |
| **Corroborate** | - Speak to others involved in the care of the person with Parkinson disease, if applicable and consented - Consult other team members to evaluate if information matches - Consider if further appointment (potentially in person) is needed |
| **Review suitability of method for future consultations** | - Reflect on how the consultation method was experienced - Revisit the preferences of the person with Parkinson disease - Consider categorizing people with Parkinson disease based on suitability for different methods of consultation |
